# The 8th edition of the American Joint Committee on Cancer tumor-node-metastasis staging system for gastric cancer is superior to the 7th edition: results from a Chinese mono-institutional study of 1663 patients

**DOI:** 10.1007/s10120-017-0779-5

**Published:** 2017-11-22

**Authors:** Xin Ji, Zhao-De Bu, Yan Yan, Zi-Yu Li, Ai-Wen Wu, Lian-Hai Zhang, Ji Zhang, Xiao-Jiang Wu, Xiang-Long Zong, Shuang-Xi Li, Fei Shan, Zi-Yu Jia, Jia-Fu Ji

**Affiliations:** 1 Key Laboratory of Carcinogenesis and Translational Research (Ministry of Education/Beijing), Department of Gastrointestinal Surgery, Peking University Cancer Hospital and Institute, Beijing, China; 2 Key Laboratory of Carcinogenesis and Translational Research (Ministry of Education/Beijing), Department of Endoscopy, Peking University Cancer Hospital and Institute, Beijing, China

**Keywords:** Staging system, Survival outcome, Gastric cancer, Chinese patients

## Abstract

**Background:**

We investigated the superiority of the 8th edition of the tumor-node-metastasis (TNM) system for patients in China with gastric cancer.

**Methods:**

The survival outcomes of 1663 patients with gastric cancer undergoing radical resection were analyzed.

**Results:**

In the 8th edition system, homogeneous 5-year survival rates among different pathological TNM (pTNM) categories belonging to the same stage were observed. However, in the 7th edition system, the differences of 5-year survival rate among pTNM categories belonging to the same stage were observed in stages IIB (*P* = 0.010), IIIB (*P* = 0.004), and IIIC (*P* < 0.001). For patients in the pT1-3 (*P* < 0.001) and pT4a (*P* < 0.001) categories, there were significant differences in survival between patients in the pN3a and pN3b categories. Furthermore, partial cases (pT4bN0M0/T4aN2M0) of stage IIIB were downstaged to stage IIIA in the 8th edition system, and the 5-year survival rate of these patients was significantly better than that of patients in stage IIIB in the 8th edition system. Similarly, the 5-year survival rate of patients in p4bN2M0/T4aN3aM0 downstaged from stage IIIC to IIIB was significantly better than that of patients in stage IIIC. Compared with the 7th edition system, the 8th edition system had a higher likelihood ratio and linear trend chi-squared score and a smaller Akaike information criteria value.

**Conclusions:**

The 8th edition system is superior to the 7th edition system in terms of homogeneity, discriminatory ability, and monotonicity of gradients for Chinese patients with gastric cancer.

## Introduction

Although the incidence of gastric cancer is declining, it remains the fourth most common malignancy worldwide, with nearly 950,000 new cases diagnosed each year [1–4]. The tumor-node-metastasis (TNM) classification by the Union for International Cancer Control (UICC) and the American Joint Committee on Cancer (AJCC) is the global standard for staging gastric cancer [5, 6]. Since the publication and application of the 7th edition TNM staging system in 2009, many studies evaluating this classification have been published [7–11]. Although most authors approve its prognostic value, some limitations exist in the 7th edition TNM staging system. There are doubts that cancer of the esophagogastric junction (EGJ), especially Siewert types 2 and 3, should be classified by the gastric staging system [12]. Another important point is regarding subgroups pN3a and pN3b. In the 7th edition staging system, separate concepts of pN3a and pN3b categories correspond to relevant stages as the only N stage as N3. In this case, the separation of pN3 stage into pN3a and pN3b does not contribute to the TNM staging system.

The 8th edition of the AJCC cancer staging manual is the most recent published edition [13, 14]. Changes in this edition from the 7th edition are mainly based upon recommendations from the International Gastric Cancer Association Staging Project [15]. The new classifications provide more comprehensive tools, including clinical (cTNM), post-neoadjuvant (ypTNM), and pathological (pTNM) classifications, for stage grouping of patients with gastric cancer under different circumstances. In the 8th edition TNM staging system, the definitions of pT and pN classification are not adjusted. Stage groupings have been reclassified. In the new staging system, pN3 is subdivided into pN3a and pN3b in stage groupings, and pT1N3b and pT2N3b are now incorporated into stage IIIB. Moreover, pT3N3b is incorporated into stage IIIC. Also, there are several changes in TNM stage groupings. For example, pT4bN0 and pT4aN2 have been downstaged from stage IIIB into stage IIIA, and pT4aN3a and pT4bN2 have been downstaged from stage IIIC into stage IIIB (Table 1).

**Table 1:** The 7th and 8th editions of the American Joint Committee on Cancer tumor-node-metastasis (AJCC TNM) staging system

7th edition	N0	N1	N2	N3
T1	IA (*n* = 245)	IB (*n* = 31)	IIA (*n* = 7)	IIB (*n* = 5)
T2	IB (*n* = 118)	IIA (*n* = 59)	IIB (*n* = 36)	IIIA (*n* = 14)
T3	IIA (*n* = 10)	IIB (*n* = 9)	IIIA (*n* = 10)	IIIB (*n* = 17)
T4a	IIB (*n* = 186)	IIIA (*n* = 211)	IIIB (*n* = 240)	IIIC (*n* = 427)
T4b	IIIB (*n* = 4)	IIIB (*n* = 4)	IIIC (*n* = 10)	IIIC (*n* = 20)

8th edition	N0	N1	N2	N3a	N3b
T1	IA (*n* = 245)	IB (*n* = 31)	IIA (*n* = 7)	IIB (*n* = 4)	IIIB^a^ (*n* = 1)
T2	IB (*n* = 118)	IIA (*n* = 59)	IIB (*n* = 36)	IIIA (*n* = 11)	IIIB^a^ (*n* = 3)
T3	IIA (*n* = 10)	IIB (*n* = 9)	IIIA (*n* = 10)	IIIB (*n* = 15)	IIIC^a^ (*n* = 2)
T4a	IIB (*n* = 186)	IIIA (*n* = 211)	IIIA^a^ (*n* = 240)	IIIB^a^ (*n* = 277)	IIIC (*n* = 150)
T4b	IIIA^a^ (*n* = 4)	IIIB (*n* = 4)	IIIB^a^ (*n* = 10)	IIIC (*n* = 14)	IIIC (*n* = 6)

^a^Changes in stage distribution in the 8th edition of the TNM staging system

These modifications are mainly based on databases from Japan, Korea, other Asian nations, and the West, with most cases collected from Japan and Korea (41.8% and 43.3%, respectively), whereas the proportion of data collected from China was much lower [15]. Therefore, the analysis was influenced by the preponderance of Japanese and Korean patients, and the validity of the 8th edition staging system for patients with gastric cancer in China is unclear.

In the light of these considerations, the purpose of our study was to investigate the validity and superiority of the 8th edition of the AJCC TNM staging system for patients in China with gastric cancer by comparison with the 7th edition of the TNM staging system.

## Materials and methods

### Patients

**Fig. 1:**
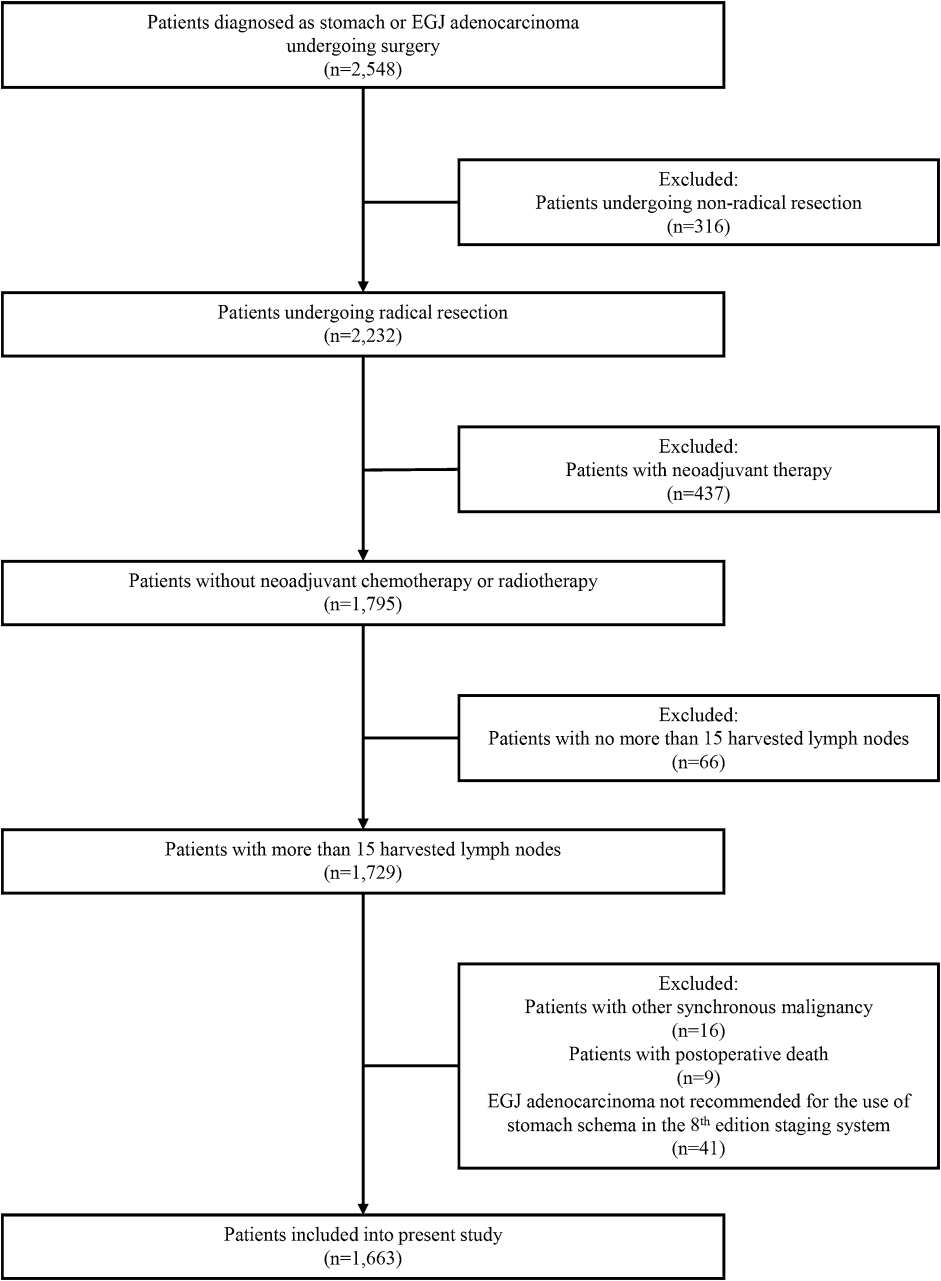
Flowchart of patient selection process in this study

This study was designed as a retrospective analysis using data from a prospectively collected database at Peking University Cancer Hospital. From January 2006 to December 2011, a total of 2548 patients with gastric cancer or EGJ adenocarcinoma underwent surgery at our hospital. Eligibility criteria included (1) gastric adenocarcinoma identified by histopathological examination, (2) no distant metastasis, (3) radical resection, (4) gastrectomy and lymphadenectomy based on Japanese Gastric Cancer treatment guidelines [16], (5) more than 15 harvested lymph nodes, (6) no other synchronous malignancy, (7) no death in the postoperative period, (8) no preoperative chemotherapy or radiotherapy, and (9) tumors located in the stomach or EGJ tumors recommended for the use of the stomach schema in the 8th edition staging system. As there was no change in the definition of stage IV between the 7th and 8th edition staging systems, patients with M1 disease were excluded from our study. Only patients with more than 15 harvested lymph nodes were included in our study to avoid staging migration caused by an insufficient number of harvested lymph nodes. Moreover, we excluded patients receiving neoadjuvant therapy because of the possibility that neoadjuvant therapy might affect pathological staging of resected specimens (Fig. 1). The purpose of our study was to compare the validity and superiority of the 8th edition staging system for Chinese patients.

A total of 1663 patients were included in the current study. The clinicopathological data collected from the database included age, sex, Lauren’s classification, tumor size, tumor location, the extent of gastrectomy, the histological depth of tumor invasion (pT), the total number of harvested lymph nodes, the number of harvested lymph nodes with histological metastasis (pN), and adjuvant chemotherapy. The terminology used in this study was based on the Japanese classification of gastric carcinoma [17]. Adjuvant chemotherapy was administered to 1363 patients who were identified as pT3/4 or metastasis in lymph nodes. Adjuvant chemotherapy was usually performed with cisplatin-based or 5-fluorouracil-based systemic therapy.

The principles of surgery were mainly based on the Japanese gastric cancer treatment guidelines [16, 18]. For tumors limited to mucosa or submucosa, D0, D1, or D1+ lymph node dissection was conducted. For lymph node-positive tumors or tumors invading to the muscularis propria or deeper, standard D2 or D2+ lymph node dissection was performed.

This study was performed with the approval of the Ethics Committee of Peking University Cancer Hospital (Reference no. 2006021). Each patient in this study signed informed consent.

### Follow-up

Follow-up was carried out by telephone interviews, e-mail communication, or outpatient reviews. Postoperative follow-up was conducted every 3 months for the first 2 years, every 6 months from the third to the fifth years, and annually thereafter until the patient died or until the last follow-up.

### Statistical analysis

All patients were restaged using the 7th and 8th editions of the AJCC TNM staging systems. Kaplan–Meier estimation and log-rank tests were performed to compare 5-year survival rate. The Cox proportional hazards regression model was used to identify independent prognostic factors by multivariate analysis. Further, the likelihood ratio *χ* ^2^ test related to the Cox regression model was used to measure homogeneity. The discriminatory ability and monotonicity of gradient assessments were measured with the linear trend *χ* ^2^ test. Further, the Akaike information criteria (AIC) within a Cox proportional hazard regression model were used to demonstrate the discriminatory ability of the given model for staging systems. A smaller AIC value indicated a more desirable model for predicting outcome [19, 20]. All statistical analyses were performed using IBM SPSS Statistics 20.0 software (SPSS, Armonk, NY, USA). *P* < 0.05 (two-sided) was considered significant in the statistical analyses.

## Results

The mean age of all patients was 61.1 (range, 20–87) years. The male to female ratio was 3.06:1. The mean number of harvested lymph nodes was 24 (range, 16–79). The median follow-up for the entire cohort was 46 months (range, 4–104). The overall 5-year survival rate of all patients was 59.0%. The majority of tumors were located in the distal two-thirds of the stomach (68.7%). EGJ tumors accounted for 7.5% of all cases. Changes in stage distribution between the 7th and 8th edition staging systems are shown in Table 1. In our study, changes of stages in the 8th edition were mostly in the subgroups of stage III. We found that the largest subgroup of stage III in the 7th edition was IIIC (*n* = 457), whereas the largest subgroup of stage III in the 8th edition was IIIA (*n* = 476). Distal gastrectomy (51.5%) was the most frequently performed operation in all cases, followed by proximal (27.1%) and total (19.8%) gastrectomy. D2 or D2+ lymphadenectomy was performed in the majority of all patients (61.8%) in our study.

### Univariate and multivariate survival analysis

**Fig. 2:**
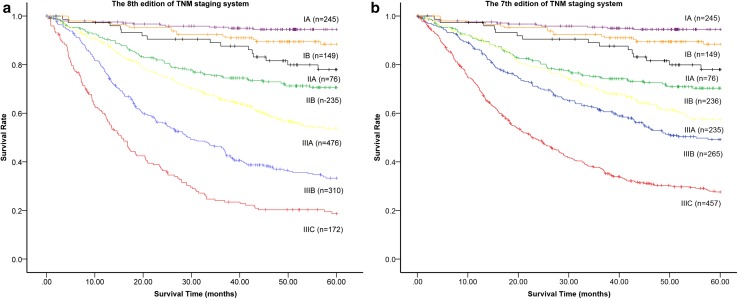
Stage-related 5-year survival rates in the 7th and 8th edition staging systems. Significant differences were observed among all stages, with the exception of stages IB and IIA (*P* = 0.131) in both 7th and 8th edition staging systems. **a** The 5-year survival rates based on the 8th edition staging system were as follows: IA: 94.5%; IB: 88.4%; IIA: 78.0%; IIB: 70.6%; IIIA: 53.8%; IIIB: 33.3%; IIIC: 18.7%. Significant differences in 5-year survival rate were observed between stages IA versus IB (*P* = 0.027), IIA versus IIB (*P* = 0.046), IIB versus IIIA (*P* = 0.001), IIIA versus IIIB (*P* < 0.001), and IIIB versus IIIC (*P* < 0.001). **b** The 5-year survival rates based on the 7th edition staging system were as follows: IA: 94.5%; IB: 88.4%; IIA: 78.0%; IIB: 70.3%; IIIA: 57.6%; IIIB: 49.2%; IIIC: 27.6%. Similarly, significant differences in 5-year survival rate were observed between the stages of IA versus IB (*P* = 0.027), IIA versus IIB (*P* = 0.045), IIB versus IIIA (*P* = 0.033), IIIA versus IIIB (*P* = 0.042), and IIIB versus IIIC (*P* < 0.001)

Survival curves according to different stages of the 7th and 8th edition staging systems were analyzed. When analyzing stage-related survival rates, significant differences were observed among all stages, with the exception of stages IB and IIA (*P* = 0.131) in both 7th and 8th edition staging systems. The 5-year survival rates based on the 8th edition staging system were as follows: IA: 94.5%; IB: 88.4%; IIA: 78.0%; IIB: 70.6%; IIIA: 53.8%; IIIB: 33.3%; IIIC: 18.7% (Fig. 2a). Significant differences in 5-year survival rate were observed between the stages of IA versus IB (*P* = 0.027), IIA versus IIB (*P* = 0.046), IIB versus IIIA (*P* = 0.001), IIIA versus IIIB (*P* < 0.001), and IIIB versus IIIC (*P* < 0.001). The 5-year survival rates based on the 7th edition staging system were as follows: IA: 94.5%; IB: 88.4%; IIA: 78.0%; IIB: 70.3%; IIIA: 57.6%; IIIB: 49.2%; IIIC: 27.6% (Fig. 2b). Similarly, significant differences in 5-year survival rate were observed between the stages of IA versus IB (*P* = 0.027), IIA versus IIB (*P* = 0.045), IIB versus IIIA (*P* = 0.033), IIIA versus IIIB (*P* = 0.042), and IIIB versus IIIC (*P* < 0.001). Potential prognostic differences among TNM categories belonging to the same stage group were investigated (Table 2). In the 8th edition staging system, homogeneous 5-year survival rates among different TNM categories were observed in each of the same stage groups. In comparison, in the analysis of the 7th edition staging system, the differences in 5-year survival rate among different TNM categories in the same stage group were observed in stages IIB (*P* = 0.010), IIIB (*P* = 0.004), and IIIC (*P* < 0.001).

**Table 2:** Comparison of prognostic differences among TNM categories in the same stage group

TNM stage	*P* value (8th)^a^	*P* value (7th)^a^
IA	–	–
IB	0.139	0.139
IIA	0.209	0.209
IIB	0.957	0.010
IIIA	0.294	0.074
IIIB	0.111	0.004
IIIC	0.179	< 0.001

^a^ *P* value calculated by Kaplan–Meier survival analysis and log-rank test

**Fig. 3:**
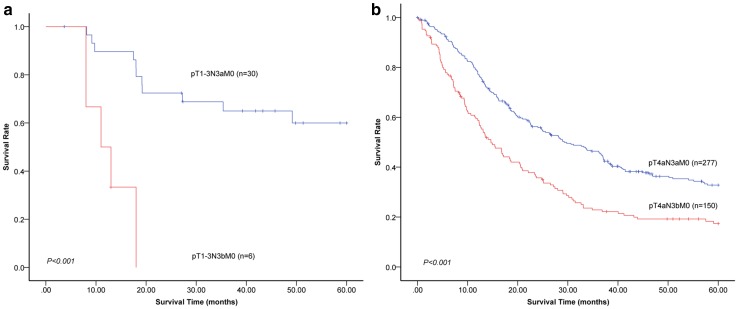
Comparisons of 5-year survival rates between patients in the pN3a and pN3b categories in the 8th edition staging system. **a** The 5-year survival rate of patients in the 8th edition pT1-3N3aM0 was significantly better than that of patients in pT1-3N3bM0 (60.0% vs. 0.0%, *P* < 0.001). **b** A significant difference in 5-year survival rate was also observed between pT4aN3aM0 and pT4aN3bM0 in the 8th edition TNM system (32.8 vs. 17.4%, *P* < 0.001)

**Fig. 4:**
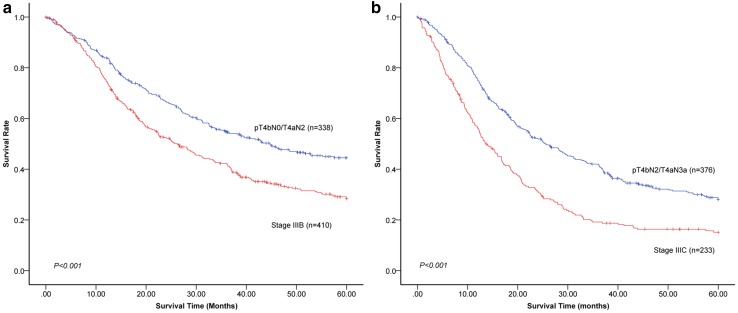
Comparisons of 5-year survival rates between patients downstaged into different stages and those in the former stages in the 8th edition staging system. **a** The 5-year survival rate of patients in the 8th edition pT4bN0M0/T4aN2M0 downstaged from IIIB into IIIA was significantly better than that of patients in stage IIIB (44.5 vs. 28.4%, *P* < 0.001). **b** The 5-year survival rate of patients in the 8th edition pT4bN2M0/T4aN3aM0 downstaged from stage IIIC into IIIB was significantly better than that of patients in stage IIIC (28.0 vs. 15.1%, *P* < 0.001)

Because pN3 are subdivided into pN3a and pN3b in stage groupings in the 8th edition staging system, prognoses between patients in the pN3a and pN3b categories were compared. Because the number of patients in the pN3 category is not large enough and the distribution of pT categories from pT1 to pT3 between patients in the pN3a and pN3b categories has no significant difference (*P* = 0.841), we conducted the analysis with a combination of pT1, pT2, and pT3 categories. The 5-year survival rate of patients in the 8th edition pT1-3N3aM0 was significantly better than that of patients in pT1-3N3bM0 (60.0% vs. 0.0%, *P* < 0.001; Fig. 3a). Similarly, significant differences in the 5-year survival rate were also observed between pT4aN3aM0 and pT4aN3bM0 in the 8th edition staging system (32.8% vs. 17.4%, *P* < 0.001; Fig. 3b). In consideration of the fact that pT4bN0M0 and T4aN2M0 were downstaged from stage IIIB to IIIA in the 8th edition staging system, prognoses of patients between the 8th edition pT4bN0M0/T4aN2M0 and stage IIIB were compared. The results showed that the 5-year survival rate of patients in the 8th edition pT4bN0M0/T4aN2M0 was significantly better than that of patients in stage IIIB (44.5% vs. 28.4%, *P* < 0.001; Fig. 4a). For the same reason that pT4bN2M0 and T4aN3aM0 were downstaged from stage IIIC to IIIB in the 8th edition staging system, prognoses of patients between the 8th edition pT4bN2M0/T4aN3aM0 and stage IIIC were compared. Similarly, the results showed that the 5-year survival rate of patients in the 8th edition pT4bN2M0/T4aN3aM0 was significantly better than that of patients in stage IIIC (28.0% vs. 15.1%, *P* < 0.001; Fig. 4b).

In all patients, nine factors were found to have statistically significant associations with overall survival in univariate analysis: age (*P* < 0.001), tumor location (*P* < 0.001), tumor size (*P* < 0.001), Lauren’s classification (*P* < 0.001), type of gastrectomy (*P* < 0.001), lymphovascular invasion (*P* < 0.001), adjuvant chemotherapy (*P* < 0.001), pT category (*P* < 0.001), and pN category (*P* < 0.001; Table 3). All the aforementioned variables were included in a multivariate Cox proportional hazards model to adjust for effects of covariates. In our study, the TNM stages in the 7th and 8th editions were highly correlated. Therefore, two separate multivariate models, one using the 7th edition and the other using the 8th edition system, were conducted. The results showed that age, Lauren’s classification, lymphovascular invasion, type of gastrectomy, and the 7th and 8th edition staging systems were all independent prognostic factors. Tumor location, tumor size, and chemotherapy did not show significant influence on multivariate analysis. The relative risk (RR) of the 8th edition pTNM staging group was found to be 3.140 for stage IB, 7.346 for stage IIA, 10.478 for stage IIB, 17.627 for stage IIIA, 28.219 for stage IIIB, and 47.097 for stage IIIC compared with stage IA. The RR of the 7th edition pTNM staging group was found to be 3.135 for stage IB, 7.322 for stage IIA, 10.335 for stage IIB, 14.747 for stage IIIA, 19.452 for stage IIIB, and 31.982 for stage IIIC compared with stage IA (Table 4).

**Table 3:** Univariate analysis of prognostic factors for survival

Variable	*N*	5-year survival rate (%)	Log rank *χ* ^2^ value	*P* value
Gender			0.193	0.660
Female	410	57.4		
Male	1253	59.6		
Age			27.083	< 0.001
≤60 years	804	66.3		
>60 years	851	52.2		
Tumor location			58.364	< 0.001
EGJ	125	48.0		
Upper	374	60.5		
Middle	341	57.1		
Lower	802	67.4		
Total	18	24.3		
Tumor size			120.002	< 0.001
≤5 cm	1175	66.7		
>5 cm	483	40.1		
Lauren’s classification			33.137	< 0.001
Intestinal	103	82.6		
Mixed	849	61.6		
Diffuse	675	54.2		
Type of gastrectomy			98.464	< 0.001
Distal	857	69.0		
Proximal	451	51.5		
Total	330	42.5		
Lymphovascular invasion			164.855	< 0.001
No	905	71.7		
Yes	732	42.3		
Extent of lymph node dissection			6.335	0.176
D0	60	56.2		
D1	172	51.4		
D1+	151	68.6		
D2	881	61.1		
D2+	147	58.0		
T category			231.597	< 0.001
T1	288	93.9		
T2	227	78.6		
T3	46	67.4		
T4a	1064	45.9		
T4b	38	34.2		
N category			451.747	< 0.001
N0	563	84.8		
N1	314	64.5		
N2	303	52.2		
N3a	321	35.7		
N3b	162	16.2		
Adjuvant chemotherapy			131.818	< 0.001
No	300	92.2		
Yes	1363	51.6

**Table 4:** Multivariate analysis of prognostic factors for survival

	*P*	RR	95% CI	
8th edition of the AJCC TNM staging system				
Age	< 0.001	1.474	1.244	1.747
Lauren’s classification	< 0.001			
Intestinal (reference)		1.000		
Mixed	0.034	1.703	1.041	2.786
Diffuse	0.002	2.199	1.342	3.603
Lymphovascular invasion	0.014	1.267	1.050	1.529
Type of gastrectomy	< 0.001			
Distal (reference)		1.000		
Proximal	< 0.001	1.462	1.203	1.777
Total	< 0.001	1.607	1.311	1.969
8th edition staging system	< 0.001			
IA (reference)		1.000		
IB	0.010	3.140	1.320	7.470
IIA	0.002	7.346	2.090	25.827
IIB	< 0.001	10.478	3.251	33.769
IIIA	< 0.001	17.627	5.571	55.767
IIIB	< 0.001	28.219	8.901	89.465
IIIC	< 0.001	47.097	14.778	150.099
7th edition of the AJCC TNM staging system				
Age	< 0.001	1.484	1.252	1.761
Lauren’s classification	< 0.001			
Intestinal (reference)		1.000		
Mixed	0.028	1.739	1.062	2.850
Diffused	0.001	2.246	1.369	3.684
Lymphovascular invasion	0.015	1.265	1.048	1.527
Type of gastrectomy	< 0.001			
Distal (reference)		1.000		
Proximal	0.001	1.391	1.146	1.690
Total	< 0.001	1.609	1.305	1.983
7th edition staging system	< 0.001			
IA (reference)		1.000		
IB	0.010	3.135	1.318	7.460
IIA	0.00	7.322	2.082	25.742
IIB	< 0.001	10.335	3.207	33.311
IIIA	< 0.001	14.747	4.607	47.207
IIIB	< 0.001	19.452	6.110	61.930
IIIC	< 0.001	31.982	10.100	101.271

*RR* relative risk

The performance of the 7th and 8th edition staging systems was assessed by the linear trend *χ* ^2^, likelihood ratio *χ* ^2^, and the AIC test. Compared with the 7th edition system, the 8th edition system had better homogeneity (higher likelihood ratio *χ* ^2^ score, 359.802 vs. 355.320), discriminatory ability, and monotonicity of gradients (higher linear trend *χ* ^2^ score, 457.014 vs. 436.618). Moreover, the 8th edition system had a smaller AIC value (8647.127 vs. 8667.523), representing better prognostic stratification.

## Discussion

The AJCC TNM staging system has undergone several major revisions in its half-century history. The 8th edition of the AJCC TNM staging system for gastric cancer incorporates several revisions to the previous edition. In our study, the stage-related survival analysis showed that the 8th edition stage grouping widened the distance between the curves, thus better stratifying survival outcomes (Fig. 2). In the multivariate analysis, the RR of the 8th edition system increased with regular intensity along with the increment of pTNM stage grouping compared with the 7th edition system. Moreover, no significant difference in survival was observed in the same stage of the 8th edition staging system. However, in the 7th edition, significant differences in survival among TNM categories were observed in stages IIB (*P* = 0.010), IIIB (*P* = 0.004), and IIIC (*P* < 0.001). Similar findings arguing that some drawbacks existed in the 7th edition staging system have previously been reported [9]. Heterogeneities probably occur because of the combination of pN3a and pN3b in the final stage grouping. All these results suggest that the 8th edition staging system is superior to the 7th edition staging system.

Additionally, for patients in the pT1–3 and pT4a categories, the 5-year survival rates of patients in the pN3a category were significantly better than those of patients in pN3b category. These results indicate that the modification designating pN3a and pN3b as separate groups in the stage grouping is reasonable. Actually, two studies have demonstrated that pN3a and pN3b should be taken into consideration in the final stage grouping [10, 15]. Furthermore, partial TNM categories (pT4bN0M0/T4aN2M0) of stage IIIB were downstaged to IIIA in the 8th edition staging system, and the 5-year survival rate of these patients was significantly better than that of patients in stage IIIB in the 8th edition (44.5% vs. 28.4%, *P* < 0.001). Similarly, the 5-year survival rate of patients in p4bN2M0/T4aN3aM0 downstaged from stage IIIC to IIIB was significantly better than that of patients in stage IIIC in the 8th edition system (28.0% vs. 15.1%, *P* < 0.001). These results demonstrate that the introduction of reclassification of some TNM categories makes the new edition prognostically more powerful.

According to the theory proposed by Ueno et al., the criteria for evaluating the performance of staging systems are as follows: (1) homogeneity within subgroups (small differences in survival among patients within the same stage), (2) discriminatory ability between different groups (greater differences in survival among patients in different stages), and (3) monotonicity of gradients shown in the association between stages and survival rates (patients with earlier stages have longer survival than those in later stages) [21]. On the basis of the foregoing results from our study, the 8th edition TNM staging system has better homogeneity, discriminatory ability, and monotonicity of gradients than the 7th edition staging system for prognostic assessment.

The main superiority of this study is that all the patients included in the current study underwent radical resection with standard lymph node dissection according to the treatment guidelines of the Japanese Gastric Cancer Association [16]. Most of the patients underwent D2 or D2+ lymph node dissection accordingly. Moreover, patients enrolled in the present study underwent adequate lymph node dissection, and the total number of harvested lymph nodes for each patient was more than 15, which prevented staging migration to a large degree. Therefore, the results of our study are reliable. However, some limitations in our study still exist. First, the definitions of clinical and post-neoadjuvant stages were included in the 8th edition of AJCC TNM staging system for the first time, and clinical and post-neoadjuvant stages were not included in the former AJCC TNM staging system. The main purpose of our study was to investigate the superiority of the 8th edition of AJCC TNM staging system by comparison with the 7th edition of AJCC TNM staging system. As a result, we did not analyze the clinical and post-neoadjuvant stages in the 8th staging system in our study. Second, the sample size remains too small, especially when stratified by the pT and pN categories, which results in low statistical power for comparison of prognosis. Further studies should be carried out to confirm our results.

## Conclusions

In conclusion, the present study demonstrates that it is reasonable to designate pN3a and pN3b as separate groups during the final stage grouping in the 8th edition TNM staging system. The reclassifications of stage groupings in the 8th edition staging system are appropriate. The 8th edition system is superior to the 7th edition system in terms of homogeneity, discriminatory ability, and monotonicity of gradients for patients with gastric cancer in China. We recommend application of the 8th edition TNM staging system for gastric cancer to patients in China.

## Abbreviations

TNM: Tumor-node-metastasis
UICC: Union for International Cancer Control
AJCC: American Joint Committee on Cancer
EGJ: Esophagogastric junction
cTNM: Clinical TNM
ypTNM: Post-neoadjuvant TNM
pTNM: Pathological TNM
AIC: Akaike information criteria
RR: Relative risk
